# Retraction: MtDNA genetic diversity and structure of Eurasian Collared Dove (*Streptopelia decaocto*)

**DOI:** 10.1371/journal.pone.0273619

**Published:** 2022-08-22

**Authors:** The PLOS ONE Editors

After this article [1] was published, questions were raised about the validity of the identification method of the dove species based on the feather samples. Additional concerns were raised about the genetic clustering of the dove species presented in [1]. In light of these issues, *PLOS ONE* reassessed this article and the genetic data provided within the article with support from a member of the *PLOS ONE* Editorial Board. Based on the outcome of this assessment, the following concerns remain unresolved:

1. Neither the original feather samples used in [1] nor images of these feather samples are available for an independent re-evaluation to confirm the identification of the dove species these feathers originated from.
2. Two independent genetic analyses of the sequences provided in [1] were performed to confirm the above identification. The neighbor-joining analysis and the maximum likelihood tree analysis carried out by a *PLOS ONE* board member suggest that the samples identified as *Streptopelia decaocto* in [1] appear to cluster with other species from the Old World Columba family, including *Columba livia*, *C*. *rupestris*, *C*. *palumbus* (Clade B in [1]), and some samples with *S*. *roseogrisea* (Clade A in [1]). Both of these analyses suggest that a subset of samples may have been misidentified and therefore the validity and reliability of results and conclusions drawn from these data are in question.

The *PLOS ONE* Editors retract this article due to concerns about the validity of the results, the reliability of the article’s overall conclusions, and the study’s compliance with the journal’s 4^th^ and 7^th^ publication criteria (https://journals.plos.org/plosone/s/criteria-for-publication). We regret that these issues were not identified prior to the article’s publication.

DL, SZ, and ZB do not agree with the retraction, and stand by the article’s findings. EAD and CE either did not reply directly or could not be reached.
